# Space-Based Observations of Plasma Waves During Conjunctions Between Host Sensors and Space Objects

**DOI:** 10.1007/s40295-026-00590-2

**Published:** 2026-05-26

**Authors:** Lauchie Scott, Paul Bernhardt, Andrew Howarth

**Affiliations:** 1 Defence R&D Canada Ottawa, Ottawa, Canada; 2 University of Alaska, Fairbanks, USA; 3 University of Calgary, Calgary, Canada

**Keywords:** Space domain awareness, Conjunctions, Plasma wave sensing, Space debris, Space object detection

## Abstract

This study describes exploratory, in-situ experimentation to measure Very Low Frequency (VLF) plasma waves (1–35 kHz) at times when a space physics satellite equipped with a radio plasma wave receiver conjuncts with other space objects. The objective was to learn if a secondary space object’s rapid passage near another satellite is detectable. If so, this would offer a new avenue to infer the presence of space debris in Earth orbit. Space objects in Earth’s ionosphere develop a region of ion density rarefactions in the wake of their orbital motion which could serve as the basis for object detection. In 2022 the Canadian space physics satellite CASSIOPE used its radio plasma physics package during conjunctions with other satellites and recorded ambient electric field data at times prior to, during and after the time of closest approach of CASSIOPE and the secondary object. CASSIOPE is designed to measure Earth’s aurora, particles, fields and has an eccentric 330 × 1200 km orbit which fortuitously samples a variety of plasma regimes in Earth’s ionosphere to test this approach. This orbit regularly crosses the altitudes of highly populated orbital shells such as Starlink, Iridium and OneWeb offering regular conjunction opportunities to attempt measurement of plasma oscillations. CASSIOPE collected electric field measurements using its crossed-dipole Radio Receiver Instrument (RRI) which detects plasma electric field oscillations. CASSIOPE sampled 35 conjunctions using the RRI from 4 March to 10 June 2022. It was surmised that if CASSIOPE traversed an ion density rarefaction the RRI should produce broadband noise at times correlating with the time of closest approach. Of the 35 conjunctions sampled, 3 exhibited VLF broadband noise somewhat correlated to the time of closest approach but were difficult to differentiate from background ambient auroral activity. One conjunction showed strong temporal correlation where a conjuncting Starlink appears to have threaded the magnetic field line between itself and CASSIOPE which also traversed the Starlink’s wake. All other conjunctions where the secondary object passed behind CASSIOPE or were quite distant (~ 5–10 km) from CASSIOPE did not show wave power exceeding the ambient background. The CASSIOPE findings indicate that sensing of ion density rarefactions in space object wakes does not appear practical to implement and clear, repeatable signatures from known space object conjunctions were not identified during this investigation.

## Introduction

Space-based optical detection of space objects is usually performed by detecting visual and infrared energy reflected (or emitted) from space objects. Missions such as the Space-based Visible (SBV) [1], Space Based Space Surveillance (SBSS) [2], Sapphire [3], NEOSSat [4], and ORS-5 [5] missions rely on this premise. These systems point optical sensors in the direction of a space object and measure sunlight reflected from their surfaces to measure their position and brightness. Space-based telescope fields of view are generally best suited to detect space objects at ranges greater than ~ 1000 km as detection of space objects in their near vicinity can become problematic. A space-based observer’s sensor’s field of view cone apex decreases the volume of space near the observer making detection of space objects in proximity (< 10 km) challenging. At these close ranges, space-based optical observers also struggle with relative angular rates > 1000 arcseconds/second making trailing loss a significant factor lessening the probability of detection at close range. These considerations make the objective of optical sensing of small space objects, less than the 10 cm size limit of the Space Surveillance Network (SSN) catalog difficult to achieve.

An alternative technique to detect space objects ‘close’ to a space-based observer is desired. While the long-term ambition would be to detect space objects smaller than the classical catalog limit of 10 cm, this paper describes experimentation where larger, “known” space objects in the satellite catalog make close approaches to a satellite operating a plasma wave receiver. This experiment is conducted to see if their passing motion is detectable as plasma oscillations. In this, we seek to answer the question: “*Can plasma waves or ion density oscillations stimulated by the motion of space objects in the ionosphere be used to infer a space object’s passage near a space-based observer?*”.

To answer this question, conjunctions between the Canadian CASSIOPE [6] satellite and other known space objects were analyzed using CASSIOPE’s radio plasma instrument. This effort was to determine if electric field oscillations that exist in the Very Low Frequency (VLF) range are measurable during conjunctions. We further analyzed specific conjunction geometries where CASSIOPE traversed the ion-acoustic wake of a secondary space object, not simply any close approach with other space objects.

This paper describes the experimental basis for in-situ measurement of Very Low Frequency (VLF) radio plasma waves in, or near, the ion-acoustic wake region of space objects during conjunctions but with a focus on CASSIOPE’s motion relative to the ion-acoustic wake of the secondary space object. A description of the sensing approach, the measurement campaign, findings, and a forward-looking view toward how to improve experimentation in this nascent area of non-traditional space situational awareness (SSA) sensing is presented in the sections below.

## Space Object Wakes

**Fig. 1:**
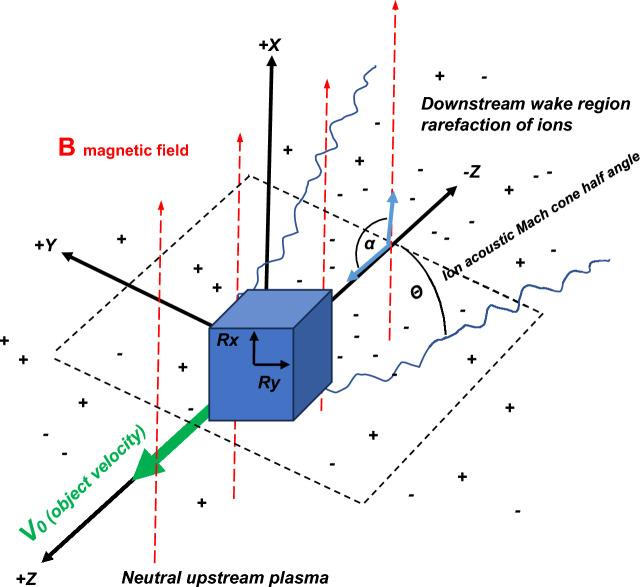
Coordinate conventions and geometric definitions for a space object immersed in a plasma with a geomagnetic field. The magnetic field (**B**) is in the *V*_*0*_*-x* plane and is shown as red dashed arrows. The space object size is radius *R*_*0*_. The angle of the magnetic field (*α*) is measured relative to the *V*_*0*_ direction (90° is shown)

The large difference between ionospheric electron and ion thermal speeds gives rise to a negatively charged wake region behind an orbiting space object [7, 8]. The motion of a space object in Low Earth Orbit (LEO) at ~ 7.5 km/s is supersonic in comparison to typical ion thermal speed (~ 1 km/s) which gives rise to a negatively charged region behind a space object (see Fig. 1). Electrons rapidly replenish the wake region behind a space object whereas the much heavier and slower ions take longer to enter the wake to reestablish plasma neutrality. This process generates ion density rarefactions, (or enhancements) in the wake behind and orbiting space object [7, 8] depending on the distance of measurement downstream in the wake.

Figure 1. shows the general wake geometry considered for this analysis relative to an object’s direction of motion relative to the geomagnetic field. We use the coordinate conventions shown in Fig. 1 where the *z*-axis is aligned with a space object’s orbital velocity vector **V**_**0**_ and the *x*-axis is perpendicular to the direction of space object motion. This coordinate frame is a rotation from the typical radial, in-track and cross-track (RIC) frame normally used in conjunction analysis but provides a useful basis bridging space object geophysical effects and the space domain awareness community’s convention for collision assessment.

The magnetic field is in the *V*_*0*_*-x* plane. The wake region of interest is in the direction of *–z* or the anti-ram direction. The orientation angle *α* of the magnetic field is measured in the *V*_*0*_*-x* plane with positive rotation about the *y-*axis. In Fig. 1 the motion of the space object is drawn perpendicular to the magnetic field (*α* = *90°)* however the magnetic field can take any orientation in the *V*_*0*_*-x* plane. If the magnetic field is aligned with the + *Z* axis, then *α* = *0°.* In Earth orbit the magnetic field can also be projected into the *y*-direction depending on the orbit analyzed.

Table 1 details the basic properties of ionospheric plasma in LEO at various altitudes helping to set the timescales and distance scales for this analysis. In LEO, the dominant charged particle densities arise from the electron [e^−^] and Oxygen [O^+^] species. At altitudes > 900 km or during nighttime conditions ionized hydrogen can exceed the density of the O^+^ species [8]. Ion and electron temperatures are assumed to be generally equal in the ionosphere. The Debye length $({\lambda }_{D})$ is the *e-folding* distance over which a plasma attenuates a Coulomb electric field. Earth’s ionospheric plasma effectively dampens electric fields over distances less than 1 cm. In our analysis the dimensions of the space objects under study are larger than ${\lambda }_{D}$ meaning that small scale electric field effects can be neglected. Similarly, the mean free path of *L*_*e*_ of electrons and ions is much larger than the size of space objects for altitudes above 100 km. Ionospheric plasma ions will directly impact a space object and either be absorbed, reflected or scattered from a space object’s surface.

**Table 1:** Properties of LEO ionospheric plasma from [7]. Bracketed values describe diurnal variation between dayside and nightside ionospheric properties

	Altitude				
Plasma properties	100 km	400 km	500 km	700 km	1000 km
*n*_*e*_ (cm^−3^)	(2–100) × 10^3^	(5–15) × 10^5^	(4–10) × 10^5^	(2–5) × 10^5^	~ 10^5^
*T*_*i*_*,T*_*n*_(K)	~ 230	1500	~ 1800	2000	3000
*n(*O^+^*)/n*_*e*_	0.95—1	0.95—1	0.3—1	0.3—1	10^–2^—10^–1^
*n(*H^+^*)/n*_*e*_	0.02—0.1	0.02—0.1	0.1—0.6	0.1—0.6	0.5—1
*L*_*e*_* (m)*	0.5	200	400	1000	8000
${\lambda }_{D}$(cm)	1	0.2—0.4	0.3—0.6	0.4—0.7	1
${v}_{th,\mathrm{e}}$(km/s)	83.5	213	233	246	301
${v}_{th}$[H^+^] (km/s)	1.9	5.0	5.5	5.7	7.0
${v}_{th}$[O^+^] (km/s)	0.5	1.2	1.4	1.4	1.8
Ω_e_ (1/s)	8 × 10^6^	7.3 × 10^6^	7 × 10^6^	6.4 × 10^6^	5.7 × 10^6^
Ω_i_ (1/s)	2 × 10^2^	1.8 × 10^2^	1.9 × 10^2^	2 × 10^2^	1 × 10^3^

Nomenclature					
*n*_*e*_	electron density	*L*_*e*_* (m)*	Mean free path	${v}_{th}$*[O* +*]*	Thermal speed, ionized O^+^
*T*_*i*_*,T*_*n*_	ion, neutral temperature	${\lambda }_{D}$	Debye length	Ω_e_ (1/s)	Electron gyrofrequency
*n(*O^+^*)/n*_*e*_	ratio of ionized O^+^ to electron density	${v}_{th,\mathrm{e}}$	Thermal speed, electrons	Ω_i_ (1/s)	Ion gyrofrequency
*n(*H^+^*)/n*_*e*_	ratio ionized H^+^ to electron density	${v}_{th}$[H^+^]	Thermal speed, ionized H^+^		

The thermal velocity of particles (${v}_{th}$) for electrons *e* and ions *i* is calculated using Eq. 1 where ${k}_{B}$ is the Stefan–Boltzmann constant (1.38 × 10^–23^ J/K), ${T}_{e}$, ${T}_{i}$ is the temperature of the electrons or ions in Kelvin and ${m}_{e}$, ${m}_{i}$ is the electron and ion mass in kg.

1 $${{v}_{th,}}_{e}= \sqrt{\frac{2{k}_{B}{T}_{e}}{{m}_{e}}} {{v}_{th}}_{,i}= \sqrt{\frac{2{k}_{B}{T}_{i}}{{m}_{i}}}$$

Two key assumptions can be made about the thermal velocity of the electrons and ion species in LEO:

1. The thermal velocity of the electrons is ~ 33 × greater than the orbital speed of a space object. Relative to electrons, the space object appears to be “standing still”.
2. The orbital velocity of the space object is greater than the thermal velocity of the ions in LEO. For ionized oxygen, the space object orbital speed is ~ 5.2 × greater than the thermal velocity. The space object is “supersonic” relative to ions in orbit.

Similar to aerodynamics, a satellite wake can be described as having a transitional boundary region similar to that of a Mach cone. The opening half angle $\theta$ of the cone is described as the ratio of the satellite’s orbital speed to the ion acoustic speed ${c}_{s}\sim \sqrt{3{k}_{B}{T}_{i}/{m}_{i}}$ and is described as

2 $$M_\text{ion acouctic}= \frac{{v}_{orbital}}{{c}_{s}}$$

and

3 $$\sin(\theta )= \frac{1}{M_\text{ion acouctic}}$$

**Fig. 2:**
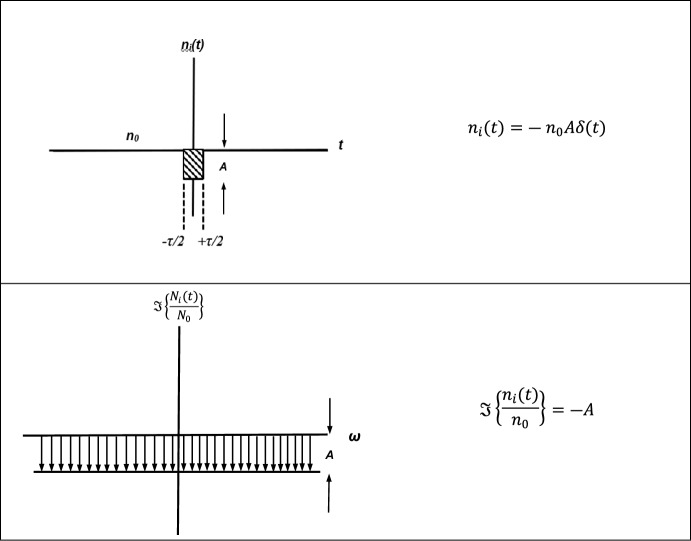
Ion density rarefaction pulse as observed by a satellite transiting the wake of another satellite. *Top*: Density and time profile of the observer transiting a sudden ion density rarefaction where the plasma has a net charge of zero, then a sudden negative charge pulse in plasma density is briefly observed. *Bottom*: broadband frequency response to a space object quickly transiting the wake of another space object

A space object immersed in ionospheric plasma with a geomagnetic field will cause ion density rarefactions in the wake direction. In this experimentation, we model the ion rarefaction as a small depression in ion density using a negative Dirac delta function (see Fig. 2.). Al’pert [7] devised an estimate of the behavior of the ion concentration in the wake of a space object by assuming the background plasma is Maxwellian distributed, and motion of the particles is governed by equations of continuity, momentum, and energy. The expression for magnetic field angles $\alpha$ slightly above zero is shown as Eq. 4

4 $$\begin{aligned}n_{i} \left( {x,y,z} \right) &= n_{0} \left\{ {1 - \frac{1}{4}\left| {\Phi \left( {\frac{{x - R_{x} }}{z\sin \left( \alpha \right) + x\cos \left( \alpha \right)}\sqrt {\frac{{m_{i} V_{0}^{2} }}{{2k_{B} T}}} } \right) - \Phi \left( {\frac{{x + R_{x} }}{z\sin \left( \alpha \right) + x\cos \left( \alpha \right)}\sqrt {\frac{{m_{i} V_{0}^{2} }}{{2k_{B} T}}} } \right)} \right|} \right. \\&\quad \left. { \times \left| {\Phi \left( {\frac{{y - R_{y} }}{{2{ \varrho }_{H} \sin \left( {\frac{{\Omega_{i} z}}{{2V_{0} }}} \right)}}} \right) - \Phi \left( {\frac{{y + R_{y} }}{{2{ \varrho }_{H} \sin \left( {\frac{{\Omega_{i} z}}{{2V_{0} }}} \right)}}} \right)} \right|} \right\}\end{aligned}$$

where ${n}_{i}$ is the spatial ion density, ${n}_{0}$ is the undisturbed ion concentration, $\Phi$ is the error function, $\alpha$ is the angle of the space object’s velocity vector relative to the background magnetic field, ${m}_{i}$ is the mass of the ion species under study, ${V}_{0}$ is the orbital speed of the space object (~ 7500 m/s), $T$ is the temperature of the ion species, $\Omega$ is the gyrofrequency of the ion in the magnetic field, ${\varrho }_{H}={v}_{th}/\Omega$ is the Lamour radius of the ions’ gyro motion, *x,y,z* is the spatial dimensions of the distribution and *R*_*x*_ and *R*_*y*_ are the *x* and *y* spatial dimensions of the object under study. Al’pert noted [7] that Eq. 4 does not account for thermal motion of the ions in the wake of a space object and that these density perturbations will merge back into the ambient background density on distance scales of size $z \sim 2\pi {V}_{0}^{2}/\Omega {\left({k}_{B}{T}_{i}/{m}_{i}\right)}^{1/2}$.

**Fig. 3:**
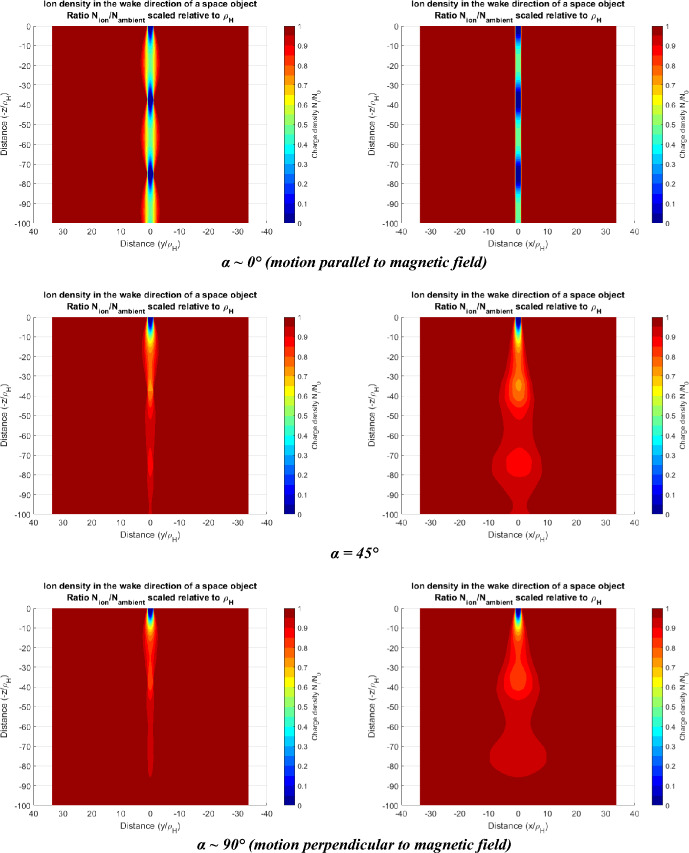
Ion density rarefactions in the wake of a space object. The object is at the top of all plots at (0,0) and shows a sharply negative plasma region directly behind the space object. The wake dimensions are scaled relative to the Larmour gyroradius ${\varrho }_{H}$ of the ions for various geomagnetic field angles *α*. The object has dimensions in the *x* and *y* directions the same size as ${\varrho }_{H}$. (*Left)*: Density rarefactions in *V*_*0*_-*y* plane which constrain the ion motion due to gyromotion constraint due to the magnetic field and forms a narrow wake in this plane. Periodic variations in density are visible in the *-z* direction. (*Right****):*** Ion density rarefactions in *V*_*0*_-*x* plane showing the periodic structure in ion density rarefactions extending in a broadened “fan-like” manner

Figure 3 shows a numerical computation of Eq. 4 sliced in the *x* and *y* axes. The appearance of spatially periodic ion density rarefactions behind a space object with wavelength of $z \sim 2\pi {V}_{0}/\Omega$ are visible. The other panels in Fig. 3 shows the extent of ion density rarefactions behind space objects for object motion nearly parallel to the magnetic field (α ~ 0°), diagonally (*α* = 45°) and transverse (*α* ~ 90°). The rarefactions in ion density form a narrow pulsating fan-like cone structure constrained within in the *y*-axis due to ion gyromotion of the particles about the magnetic field lines (see Fig. 3 left column). In the *x*-direction the ions move parallel to the magnetic field and are not affected by the gyromotion constraint, and their thermal behaviour governs their motion. The expansion fan of these ion density rarefactions in the *x*-direction depends on the size of the object relative to the gyro radius of the ions and the thermal velocity of the particles. At the altitudes of interest for this study the wake region should extend approximately ~ 1500 m behind a space object in the lower altitude (< 500 km) O + ion dominant environment. At higher altitudes (> 800 km) the wake should extend ~ 250 m behind an object corresponding to the H^+^ dominant environment. The magnitude of the ion density rarefactions varies from a fraction of a percent to ~ 10% depending on the orientation of the magnetic field relative to the object motion.

If an observer spacecraft were to “conjunct” through this ion density rarefaction fan, the charge density can be estimated using Gauss’ law by measuring the divergence of the electric field.

5 $$ \nabla \cdot\mathop{E}\limits^{\rightharpoonup} = \frac{1}{{ \in_{0} }}\rho \left( {x,y,z,t} \right) $$

where ${\epsilon }_{0}$ is the permittivity of free space (8.854 × 10^–12^ C/(V·m)) and $\rho$ is the net charge density (C/m^3^). Electric field perturbations due to time-spatial variation in plasma density should be detectable during a space object’s rapid passage through the wake region of another space object. Noting that the ion density rarefaction varies from 0.1–10% in Fig. 3 makes this wake region plasma slightly negative depending on the location of the observer’s motion through the wake region of a conjuncting satellite. Taking Gauss’s law in one dimension using Eq. 5 we obtain.

6 $$\frac{dE}{dr}= \frac{1}{{\epsilon }_{0}}\rho \left(x,y,z,t\right)\to \frac{dE}{dt}=\frac{1}{{\epsilon }_{0}}\rho \left(x,y,z,t\right)\frac{dr}{dt}=\frac{1}{{\epsilon }_{0}}\rho \left(x,y,z,t\right){V}_{rel}$$

Taking for Fourier transform of both sides of the time varying electric field expression in Eq. (6) and assuming that the spatial density rarefaction is modelled as a negative Dirac delta function (Fig. 2), multiplying by *q* (electron charge (1.602 × 10^–19^ C) and replacing the net charge density $\rho$ with *n*_*0*_ representing the ambient electron and ion density

7 $$-i\omega E\left(\omega \right)=\frac{1}{{\epsilon }_{0}}{V}_{rel}\mathfrak{I}\left\{{qn}_{i}\left(t\right)\right\}\to E\left(\omega \right)=\frac{{qV}_{rel}}{-{i\omega \epsilon }_{0}}\mathfrak{I}\left\{{n}_{0}\left(-A\delta (t)\right)\right\}=\frac{{-iqn}_{0}{V}_{rel}}{{\omega \epsilon }_{0}}\left( A(1)\right)$$

The complex power of Eq. 7 indicates that broadband noise should be observed proportional to the magnitude of rarefaction percentage *A* and the magnitude of the relative velocity of the objects *V*_*rel*_ during an observer’s motion through the wake of a space object. The power should exhibit a “fall off” inversely proportional to the angular frequency ~ $1/\omega$. The absolute magnitude representing the ‘power’ of Eq. 7 is

8 $$ \left| {E\left( \omega \right)} \right|^{2} = \frac{{\left( {qN_{0} V_{rel} } \right)^{2} }}{{ \in_{0}^{2} }}\frac{{A^{2} }}{{\omega^{2} }}\left[ {{\mathrm{V}}^{2} /{\mathrm{m}}^{2} } \right] $$

At the altitudes of this study, with average orbital speeds of ~ 7.5 km/s, a 1% ion density rarefaction in the wake of a space object should generate broadband electric field amplitudes on the order of ~ 8 V/m at 1 kHz, ~ 1.4 V/m at 6 kHz and ~ 0.8 V/m at 10 kHz.

For completeness, we examine the magnitiude of the magnetic field perturbation from a moving, charged space object to compare the viability to sense it using a magnetometer. The magnetic field deviation for a moving point charged object can be estimated from the Biot-Savart law

9 $$ \mathop{B}\limits^{\rightharpoonup} = \frac{{\mu_{0} }}{4\pi }\frac{{q\mathop{V}\limits^{\rightharpoonup} \times \mathop{r}\limits^{\rightharpoonup} }}{{r^{3} }} \left[ {\mathrm{T}} \right] $$

where ${\mu }_{0}$ is the magnetic permittivity of free space (4π × 10^–7^ N·s^2^/C^2^), $\mathop{V}\limits^{\rightharpoonup}$ is the relative velocity of the object and $\mathop{r}\limits^{\rightharpoonup}$ is the position vector of the observer.

The potential for an orbiting LEO space object is estimated to be approximately – 0.45 V due to the balance of relative ion and electron currents impacting the body [8]. We further presume the object stores charge as a 1-m diameter spherical object with capacitance of ~ *2πε*_*0*_*D.* Assuming orthogonal motion between the space objects, a relative velocity of ~ 10,000 m/s, and a closest approach of 10 m to the observer the peak magnetic field perturbation is of the order of 2.5 × 10^–7^ nT. This is considerably below the resolution for CASSIOPE’s magnetometer (0.0625 nT) and several orders of magnitude smaller than the resolution for magnetometers used for satellite attitude estimation (~ 1–2 nT). While sensing magnetic field perturbations due to the motion of a charged object does not appear viable in LEO it is possible that other wave modes may be excited (see Sect. 8). Conversely, some conceptual work to sense space objects in geostationary orbit has been examined using this principle. Due to the higher charge accumulation on geostationary satellites having potentials ~ 100–500 V there may be viability to sensing them in that specific plasma environment [9].

## CASSIOPE

**Fig. 4:**
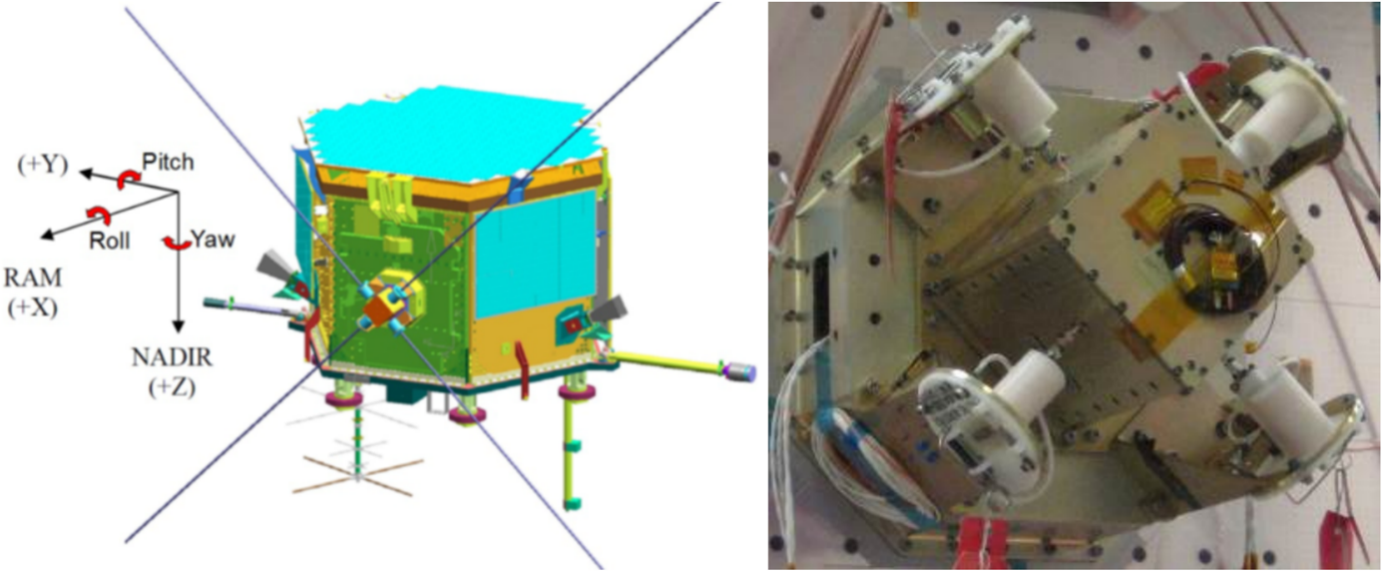
*Left:* CASSIOPE satellite with its four monopole antennas deployed. *Right:* RRI with antennas stowed. Image credit: University of Calgary [11]

The sensing platform used in this experiment was the CAScade, Smallsat and IOnospheric Polar Explorer (CASSIOPE) a Canadian space-physics mission (see Fig. 4.) launched in September 2013 to perform measurements of Earth’s geospace environment using its e-POP space physics package [6]. CASSIOPE is in a 330 × 1150 km orbit (as of 2023) at 81° inclination and registered with a COSPAR identification as 2013-055A. CASSIOPE is operated by the University of Calgary with Earth stations in Kiruna (Sweden), Inuvik (Canada), and O’Higgins (Antarctica). The instruments aboard CASSIOPE enable ionospheric and auroral research by collecting magnetic, electric field, GPS signal strength, near infrared optical, and ion data. CASSIOPE’s last reaction wheel ceased functioning in 2021 and is now in a Sun-aligned spin which keeps sunlight on its main solar panel. CASSIOPE now relies on star tracker inputs for attitude estimation and its magnetorquers for coarse attitude control of its yaw spin rate of ~ 1°/s. This spin rate enables some e-POP science instruments to continue data collection despite CASSIOPE’s diminished attitude control state.

**Fig. 5:**
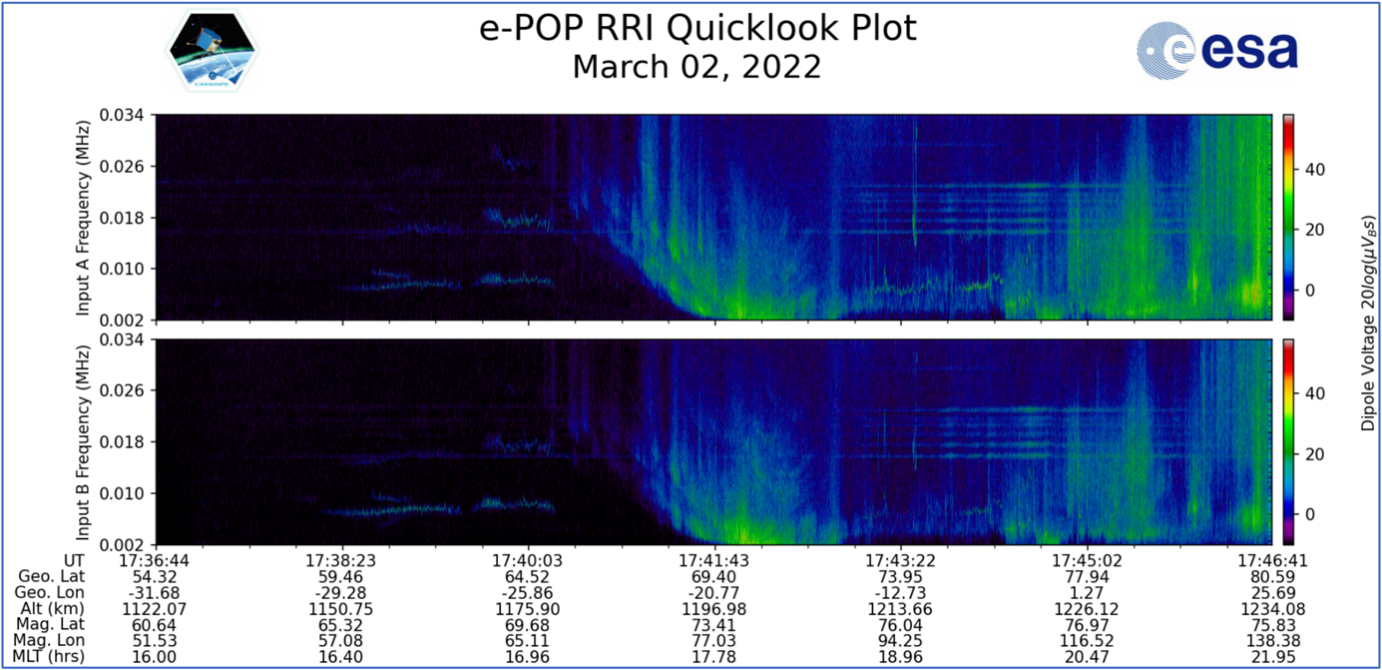
Sample electric fields spectrogram from CASSIOPE’s RRI on 2 March 2022 during a pass through the auroral region near 70° north latitude. Image credit University of Calgary [11]

Measurements of electric field oscillations are achieved using CASSIOPE’s Radio Receiver Instrument (RRI). The RRI was designed to measure the morphology and dynamics of ionospheric F-region density structures, auroral wave-particle interactions and backscatter [10]. The RRI uses four monopole antennas which measure electric field strength on a variety of channels and bandwidths. These antennas can be operated as individual monopoles or as dipoles (see Fig. 4. Left). The antennas are each 3 m long which measure electric fields from 1 µV/m to 1 V/m at a sample rate of 62,500 samples per second. The RRI can measure electric field frequencies spanning 10 Hz to 18 MHz. The crossed dipole arrangement provides 2-dimensional polarization insight such that wave direction can be inferred. A spectrogram of detected electric field data based on the in-phase and quadrature measurements from the antennas working in dipole mode is shown in Fig. 5 where CASSIOPE was traversing high latitude and observing significant Very Low Frequency (VLF) energy while transiting the auroral region. Given that CASSIOPE would see electric field strengths exceeding 1 V/m at frequencies less than 6 kHz, it is expected that the RRI would saturate at lower frequencies if a satellite wake traversal were to occur.

## Experiment Planning

Planning this collection campaign required very low overhead and infrastructure but did need to respect the memory limitations of CASSIOPE. The RRI does not record continuously and is activated for short time intervals (~ 10 min) when CASSIOPE traverses geophysical regions of interest. This is due to the high memory demands of RRI operation which samples at ~ 62 kHz [10].

The time of CASSIOPE conjunctions were forecasted using the Celestrak SOCRATES [12] website. SOCRATES’ spherical 5 km radius screening volume enabled rapid planning of RRI data collects early in this effort. SOCRATES uses two-line orbital elements to predict conjunctions where the orbital positions are accurate to a few kilometers during prediction. The 18th Space Control Squadron’s Conjunction Data Messages (CDMs) were also used to plan RRI operation. CDMs use the Special Perturbation orbital catalog which tends to be accurate from ~ 100 m to 500 m in LEO. CDMs use an ellipsoidal screening volume of dimensions 0.4 km radial, 44 km in-track and 51 km cross-track [13]. CDMs conjunctions provide 2–3 days of lead-time for commands to be uploaded to CASSIOPE’s RRI instrument to begin recording electric field measurements centered on the Time of Closest Approach (TCA). For each planned conjunction using either using SOCRATES or the 18th’s CDM service, a 10-min data collection centered on the conjunction’s TCA was programed on CASSIOPE’s flight computer. Time-tagged commands for RRI recording were generally uploaded a day or so before the conjunction during one of CASSIOPE’s daily ground station passes.

**Fig. 6:**
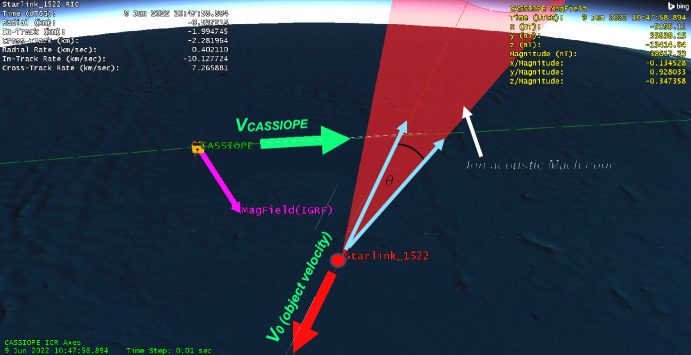
Geometry for a conjunction where the motion of CASSIOPE passes through the ion-acoustic Mach cone of the secondary space object in LEO

In addition to either SOCRATES or 18th CDM messages, a further criterion was applied where a 6 km long conical region behind the secondary object’s -*V*_*0*_ direction was added. A 10° half angle conical region would test if CASSIOPE would traverse the area in the space object wake (see Fig. 6). This conical region represents an approximation of the ion acoustic Mach cone geometry. This screening criteria significantly reduced the number of potential conjunctions as the Mach cone is a much more restrictive screening geometry compared to SOCRATES or the 18th’s conjunction criteria.

CASSIOPE’s RRI was set to acquire electric field measurements at its usual sampling rate of 62,500.33933 Hz [10]. If CASSIOPE were to transit an ion depleted region of ~ 1 ion gyroradius (~ 5 m) width at an average speed of 7.5 km/s the RRI would acquire approximately 41 electric field samples during its traversal. VLF frequencies in the 10 Hz to 36 kHz regime was chosen for this experimentation due to the likelihood of higher broadband power at lower frequencies. A + 18 kHz offset to spectrogram data collected in the VLF range is applied when operating the RRI to correct its collection bandwidth to the frequency range being measured [10]. After the conjunction CASSIOPE’s RRI data are downloaded two days later and processed into spectrograms by the University of Calgary’s e-POP data server.^1^ At this stage, the data are further analyzed for the presence of wave energy prior to, during, and after the conjunction. RRI data are also available for analysis with separate applications by downloading h5-formatted binary files [11] available from the University of Calgary e-POP data server.

## Measurements and Results

From March to June 2022, 35 conjunctions were sampled using the RRI. Appendix 1 details the space objects which had close approaches with CASSIOPE and RRI data collected. It should be noted that a variety of miss distances and close approach trajectories were attempted in this timeframe to determine if wave effects could be correlated with the secondary object’s passing. Many conjunctions acquired data at very large distances to determine if effects, other than wake density rarefactions, could be measured.

**Fig. 7:**
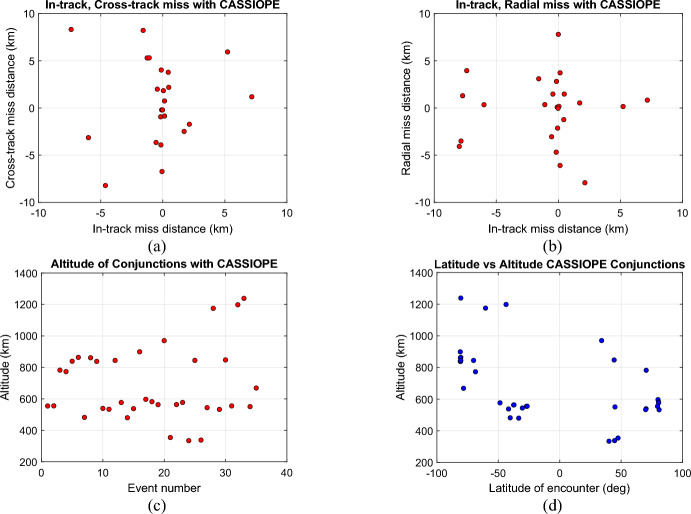
**a** In-track, cross-track miss of space objects relative to CASSIOPE **b** Radial, in-track miss distances **c** altitude of the conjunctions **d** latitude-altitude plot

Figure 7a–c shows the distribution of radial, in-track and cross-track miss distances and the altitudes that the conjunctions were observed by CASSIOPE. It is notable that a large portion of the conjunctions occur at ~ 550 km and 800 km altitudes reflecting the two most heavily populated orbital shells in LEO. Other conjunctions above 1000 km are likely to have encountered plasmaspheric ions (primarily H^+^) where the wake region is likely to be short (~ 250 m) behind the space object. Figure 7d shows that most of the conjunctions occurred at mid-latitudes or higher, suggesting the magnetic field was somewhat perpendicular to the direction of space object motion at TCA.

**Fig. 8:**
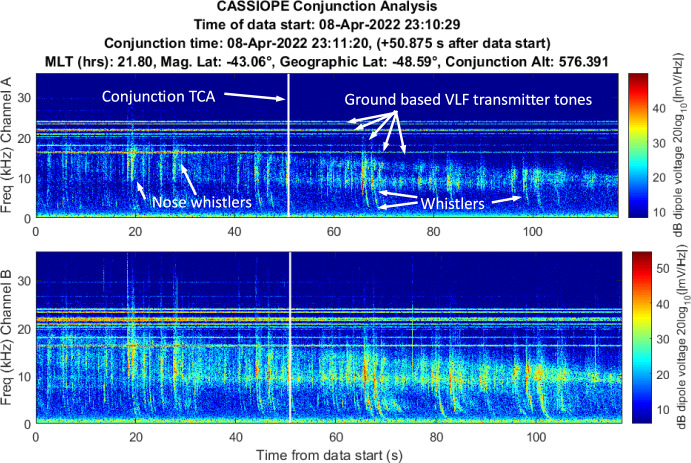
Spectrogram of detected VLF electric fields recorded by CASSIOPE’s RRI dipoles Channel A: dipoles 1,2 and Channel B: dipoles 3,4 during a distant pass between CASSIOPE and Starlink 2572 (miss distance was ~ 95 km). There was no expectation to see any detectable signal from the Starlink during the passage and representative artificial and natural noise sources are indicated

Figure 8 shows an example spectrogram of CASSIOPE RRI channel A and B electric field measurements collected from the dipoles during a very distant pass of a Starlink satellite (~ 95 km). This conjunction served as a calibration reference where no ion density rarefaction was expected to be observed and provides an overview of the characteristics of a VLF spectrogram’s natural and artificial background signatures. The spectrograms were processed over the frequency range of 200 Hz–36 kHz. The conjunction is demarcated by a vertical white line which indicates the conjunction TCA.

Artificial and natural noise sources are identifiable in Fig. 8. Ground-based VLF transmitters are visible as horizontal constant “tones” at 16, 18, 20, 22, 24, 25 kHz. These frequencies are used for subsurface naval communications and time reference. Below 10 kHz several natural ionospheric “whistlers” [14] are visible where terrestrial lightning impulses travel along Earth’s magnetic field lines, reflect at the ionospheric boundary and travel backward to the conjugate ionospheric boundary. Whistlers last for 1–2 s, and the velocity of the low frequency waves travel slower than the high frequencies. This creates a whistler’s unique “descending-whistle” tone when played as a sound. “Nose whistlers” are also shown where lightning directly couples from the ground to space and has broadband power above 6 kHz. It is also notable that in CASSIOPE’s Channel B (Fig. 8 lower plot) whistlers and other noise sources are stronger in this polarization suggesting the CASSIOPE’s dipoles 3 and 4 were more aligned with the waves’ polarization at the time of measurement. No wave energy is attributed to the passing Starlink satellite in this plot as the separation between CASSIOPE and the passing Starlink was ~ 95 km nor particular power is shown at the TCA. We now describe the findings from the conjunctions between CASSIOPE and other space objects during the test campaign.

Four conjunctions of 35 showed wave power near the time of closest approach and are presented in the following subsections. The highlighted conjunctions are ordered in the time that they were observed and a short description of the degree of association between the wave activity and the conjunction is identified along with the secondary object’s name.

### Starlink 2672 (Weak Association)

**Fig. 9:**
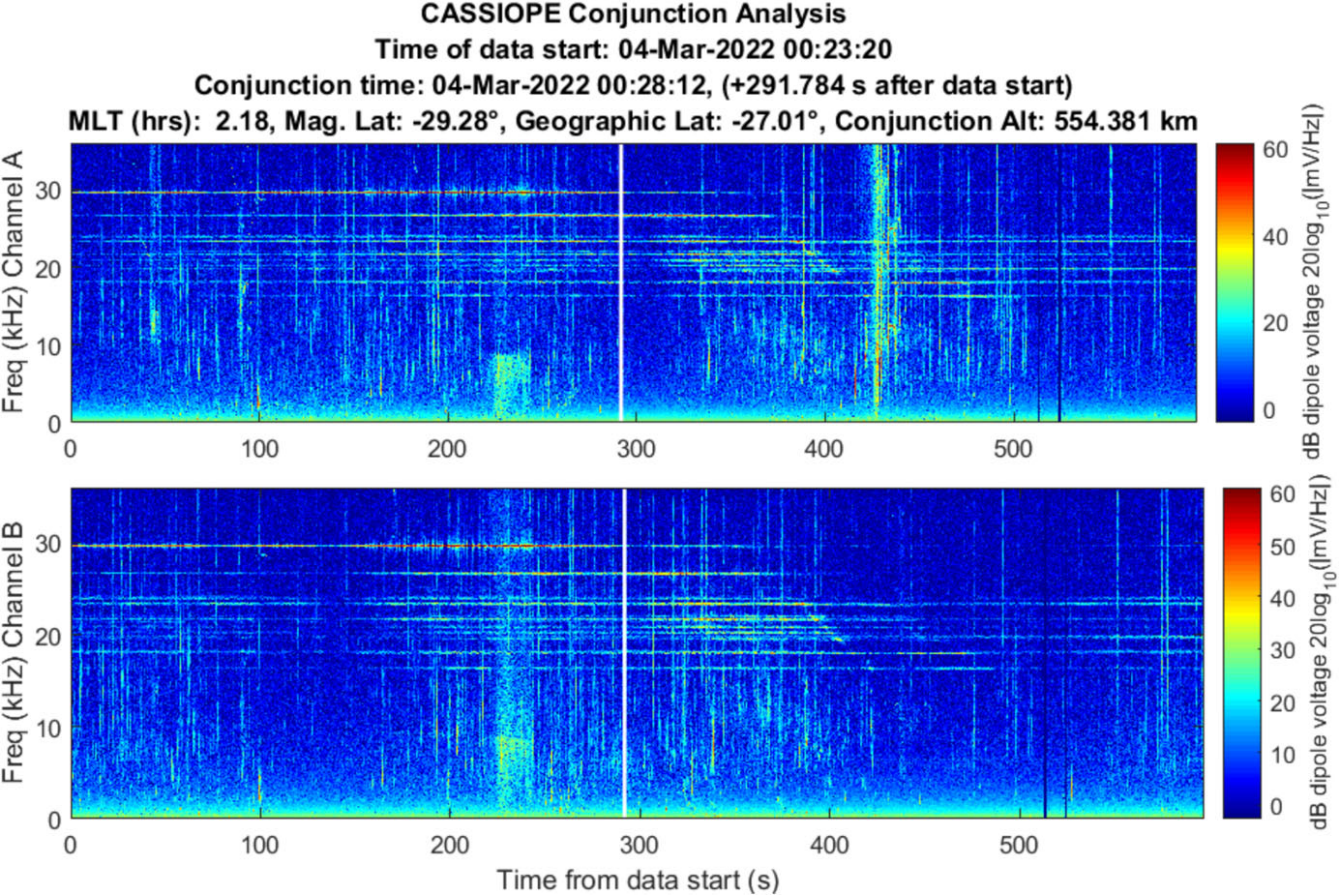
Spectrogram of CASSIOPE’s conjunction with Starlink 2672. Miss distance of 17.7 km. Note that a broadband burst of energy at low frequencies is detected, but ~ 63 s “earlier” than the expected time of conjunction

Starlink 2672 was predicted to miss CASSIOPE by 17.7 km with an in-track miss distance of -7.71 km. Starlink 2672 was predicted to pass well behind CASSIOPE and it would not pass through the Starlink’s wake. The RRI was activated anyway, and the results are shown as Fig. 9. Some broadband energy at lower frequencies was detected, but nearly 63 s prior to TCA and appears at the 229 s mark. Channel B appears to show more wave energy in that polarization. The energy appears to be “capped” by the Lower Hybrid Resonance frequency (LHR) which is the mix of the plasma frequency and the electron cyclotron frequency. An examination of the satellite catalog at the time of the burst did not show other space objects in the immediate vicinity or threading the magnetic field vector through CASSIOPE. It is not known if the electric propulsion system was active on Starlink 2672 at the time that the RRI was recording its data. A nose whistler appears at the 420 s mark and does not appear correlated with the conjunction. The wave power shown in the spectrogram is weakly associated with the TCA as the broadband power shown on Channel B is nearly 60 s prior to the conjunction itself. The conjunction is considered to be weakly associated with the wave power shown in Fig. 9.

### Starlink 2521 (Stronger Association)

**Fig. 10:**
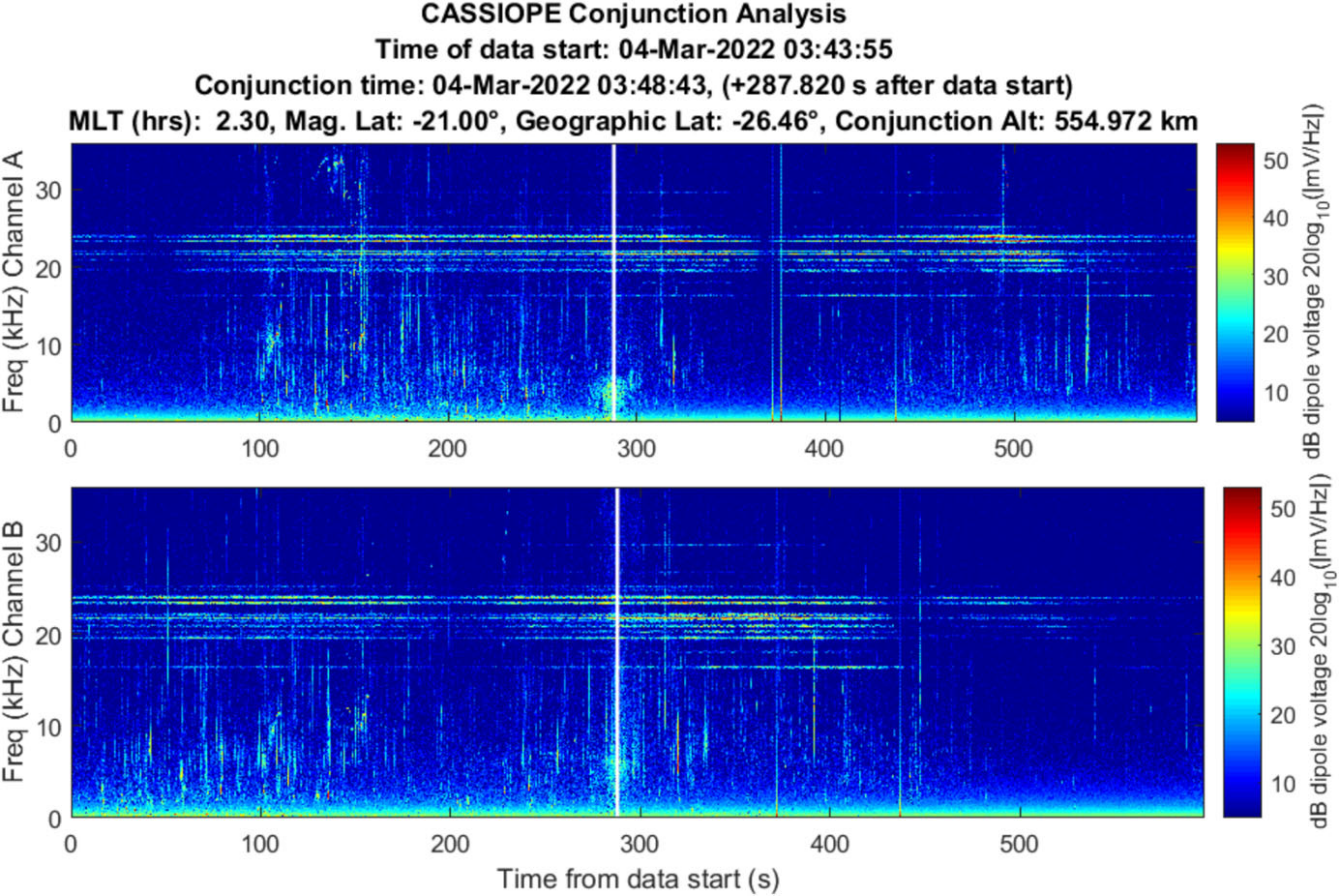
Spectrogram of detected VLF electric fields during CASSIOPE’s conjunction with Starlink 2521 on 4 Mar 2022. Miss distance was 0.24 km. A broadband burst of power at low frequencies is observed centered on the TCA of the conjunction but does not show much power at frequencies above the LHR (~ 6 kHz)

Starlink 2521 was predicted to miss CASSIOPE by 0.24 km with an in-track miss distance of – 0.090 km based on the publicly available orbital catalog data. CASSIOPE was predicted to pass ahead of Starlink 2521 during a night-side pass between the two objects. RRI data for this close approach is shown in Fig. 10. Broadband energy at frequencies less than 10 kHz was detected centered on the conjunction and with most power at the LHR. Channel B appears to show more wave energy in that polarization. A communication with SpaceX [15] indicated that Starlink 2521 was not using its electric propulsion system at the time this RRI data were recorded.

**Fig. 11:**
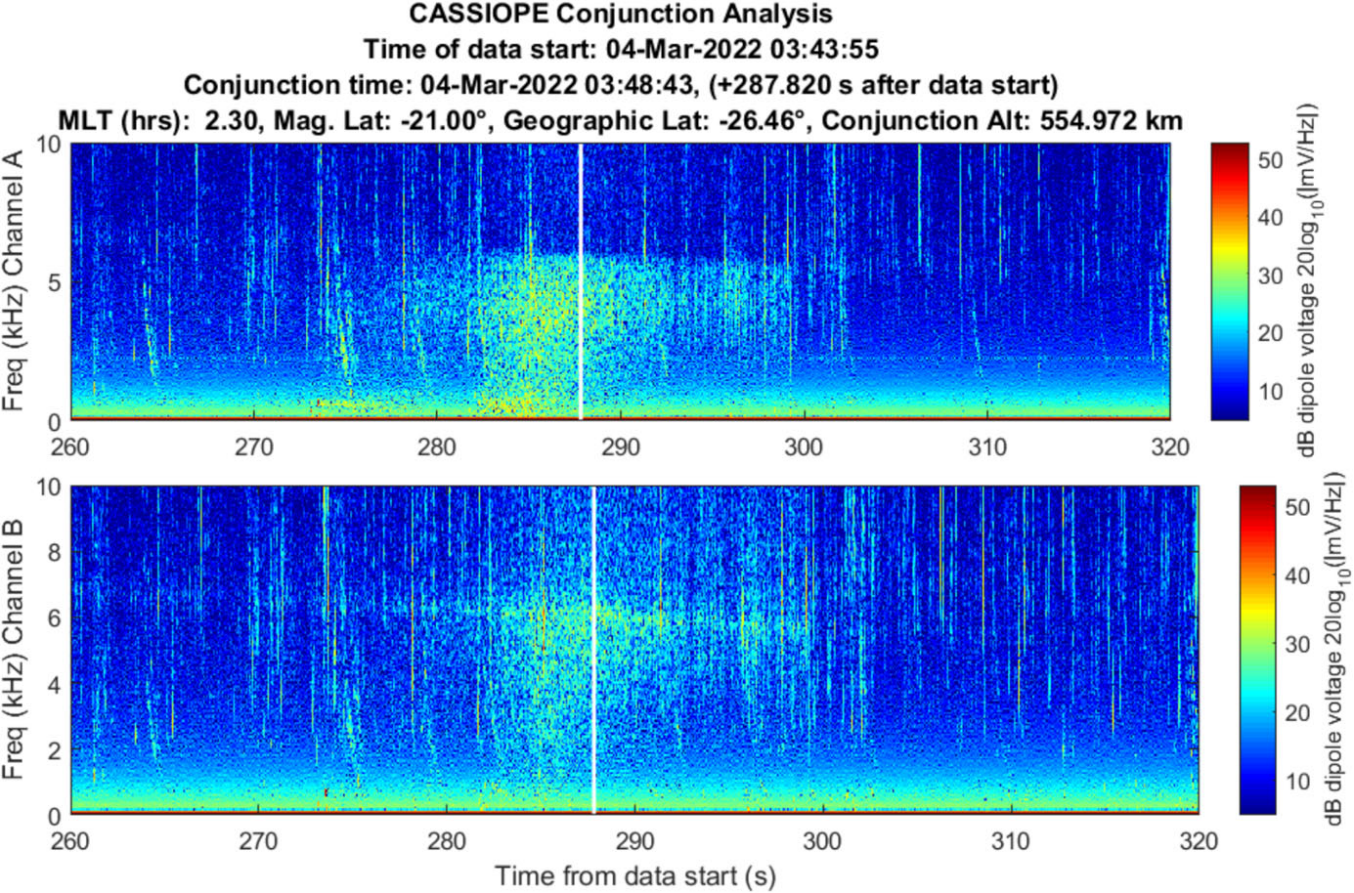
Spectrogram of detected VLF electric fields during CASSIOPE’s conjunction with Starlink 2521 with an emphasis on the time of conjunction and showing power below 10 kHz frequencies

The burst of power is emphasized in a zoomed in plot in Fig. 11 but appears to be “capped” by the LHR of ~ 6 kHz at the time on conjunction. There is a strong pulse of power about 3 s prior to TCA. The time width of the pulse is ~ 17 s which is longer than anticipated for a broadband noise spike. While well correlated in time for the conjunction, it appears that other dynamics were driving the broadband power during this conjunction based on the overall 17 s duration of the event.

**Fig. 12:**
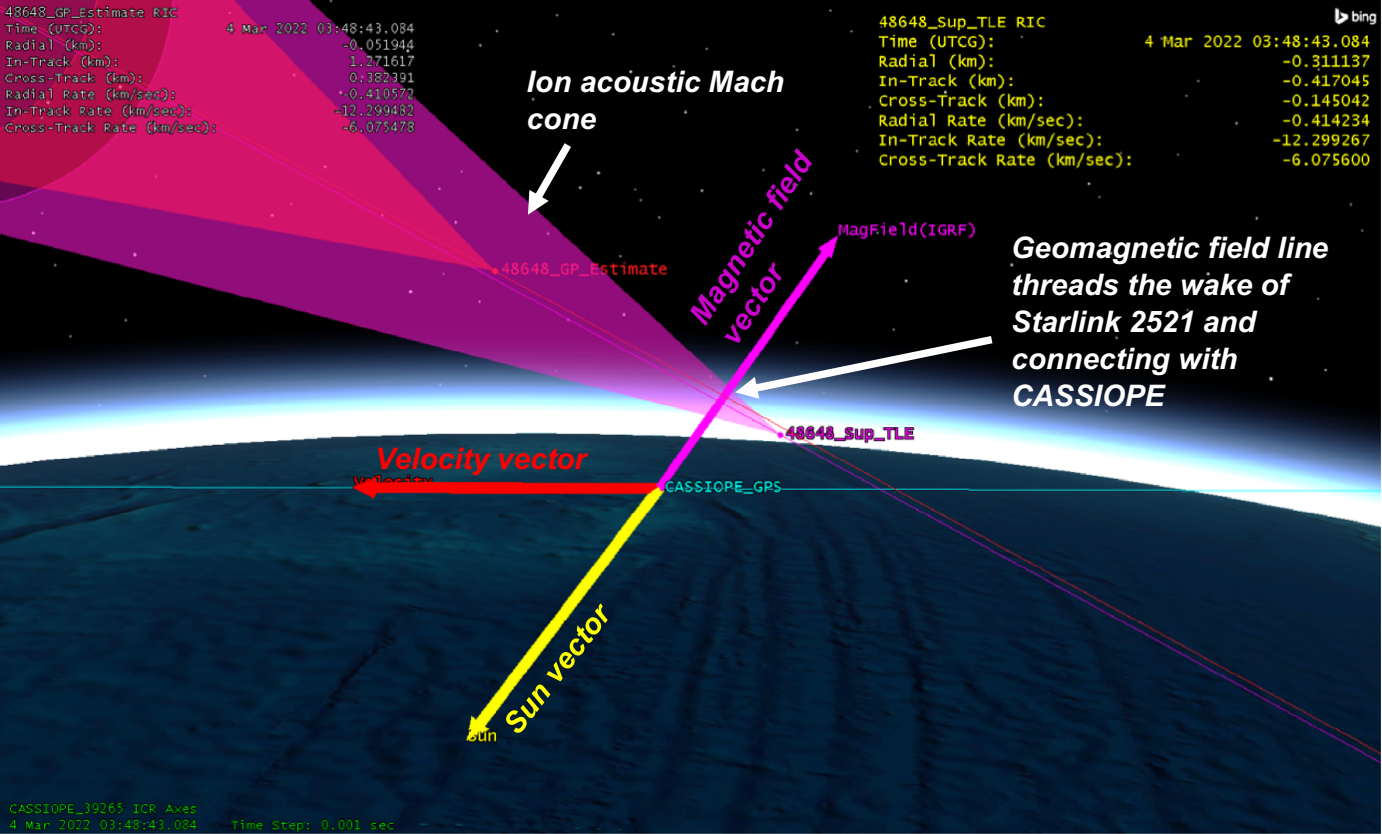
Geometry of the conjunction between CASSIOPE and Starlink 2521. The supplemental TLEs available from Celestrak [12] are ~ 2 km further in-track compared to the elset available from the satellite catalog. The geomagnetic field vector “threaded” the wake region when the objects were separated by − 417 m in the in-track direction

Given the close approach between the two objects, further orbital analysis was performed. CASSIOPE’s GPS reference ephemeris was obtained yielding ~ 5-m precision in its orbital position. Supplemental TLEs available from Celestrak [12] for the Starlink satellite were used to further refine its orbital position. These supplemental TLEs which are “fitted” on Starlink’s operator ephemeris and are generally precise to ~ 240 m at epoch. This refined orbital data indicated that Starlink 2521 flew somewhat above CASSIOPE during the conjunction by ~ 311 m, but the geomagnetic field vector which extended through CASSIOPE “threaded” Starlink 2521’s wake during conjunction (see Fig. 12.). This geometry suggests that electrostatic noise [16, 17] may be the cause of this effect, and not the ion density rarefaction that was envisaged for this experiment. Section 8 describes the historical basis of this effect where other space physics missions have observed broadband noise from the wake of moving space objects.

### COSMOS 2251 Debris (Medium-Weak)

**Fig. 13:**
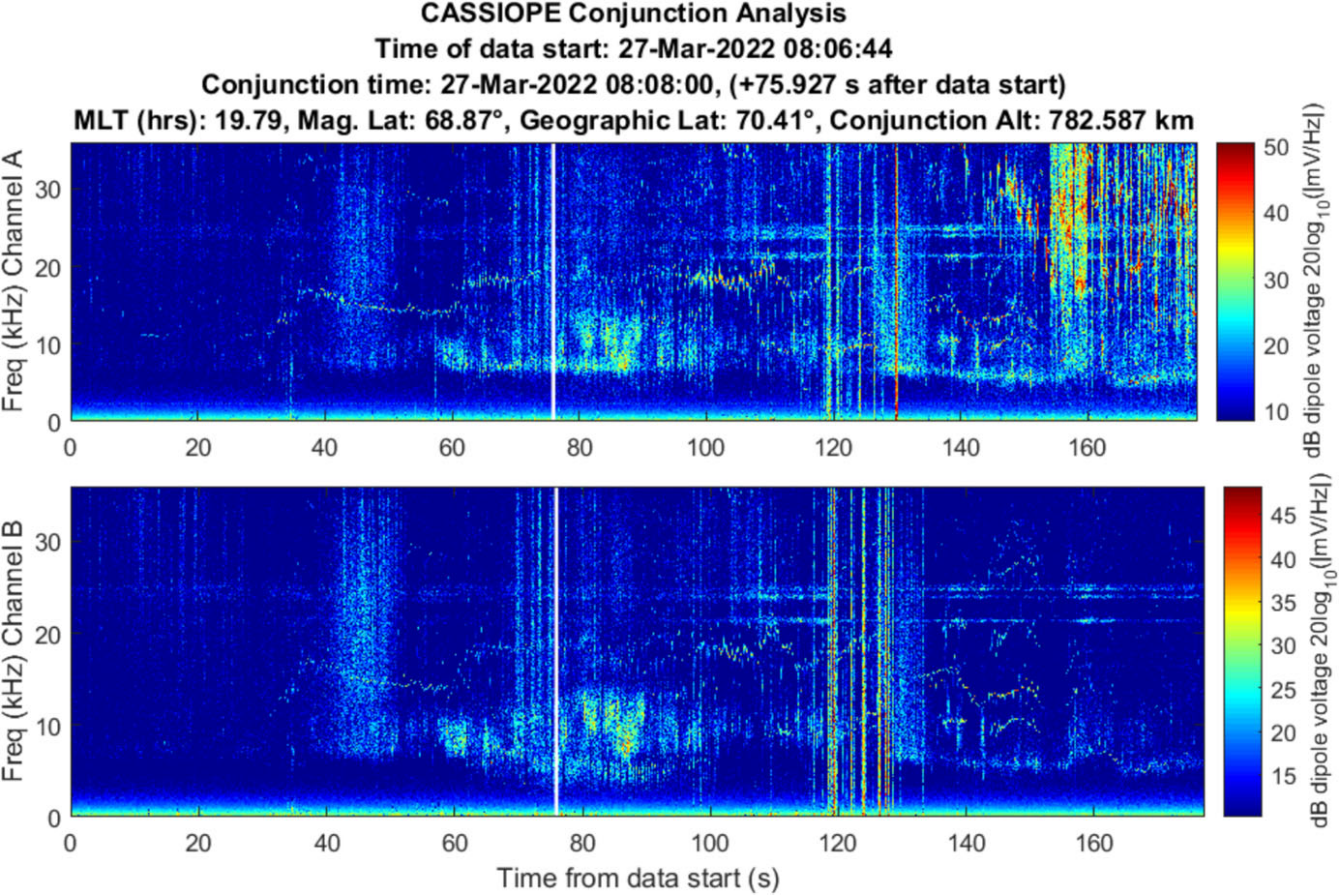
Spectrogram of detected VLF electric fields during CASSIOPE’s conjunction with COSMOS 2251 debris

**Fig. 14:**
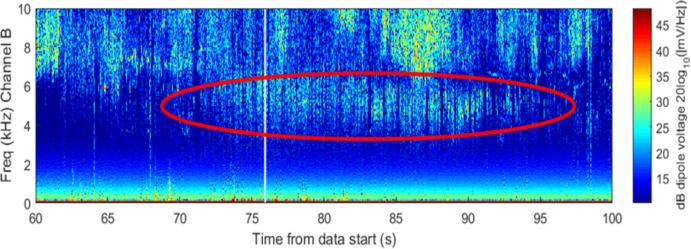
Spectrogram of detected VLF electric fields during CASSIOPE’s conjunction with COSMOS 2251 debris emphasizing Channel B energy detected under 10 kHz. A burst of power is visible from 4 to 6 kHz

**Fig. 15:**
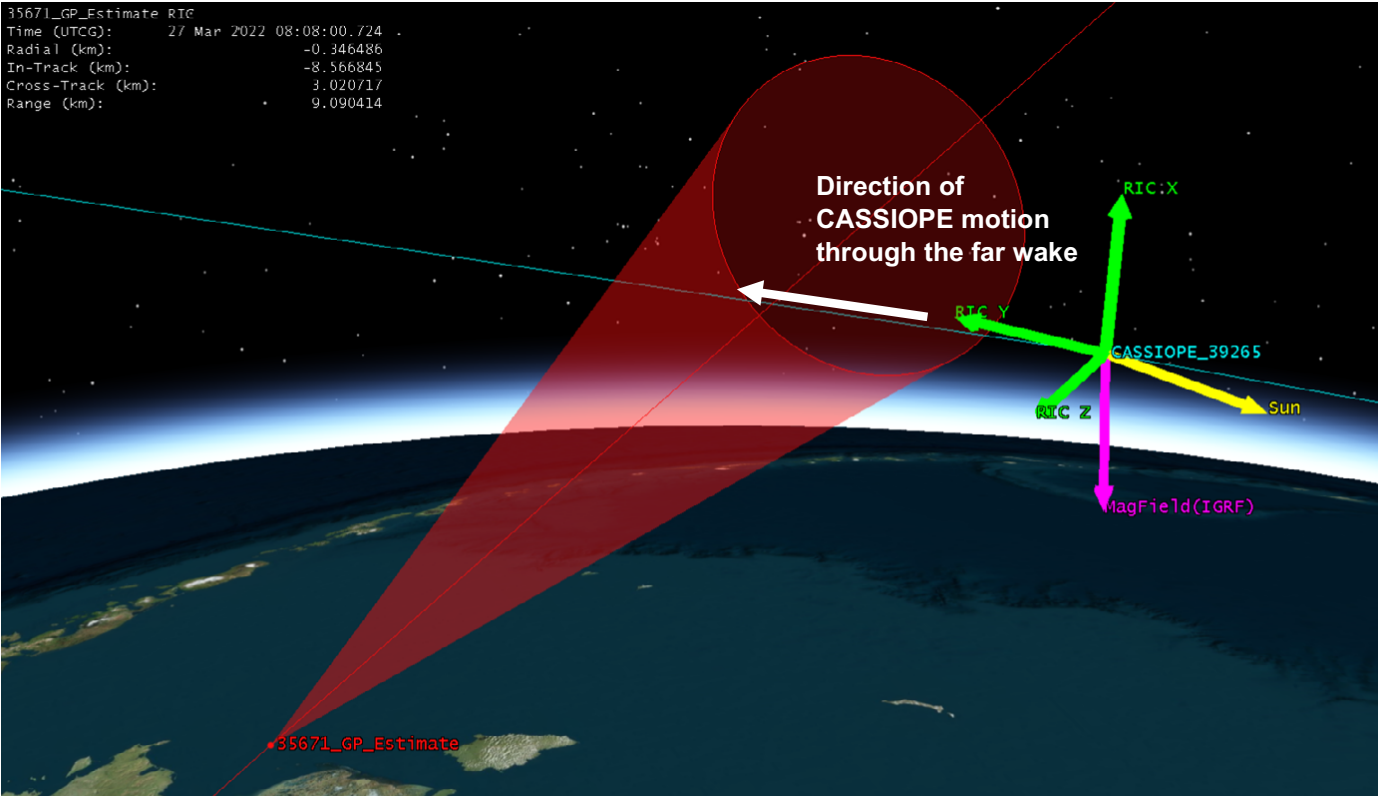
Motion of CASSIOPE toward the wake region of the COSMOS 2251 debris. CASSIOPE’s pass is well behind the debris object and rapidly traverses the far wake

A piece of COSMOS 2251 debris was predicted to miss CASSIOPE by 7.92 km with an in-track miss distance of 5.95 km. RRI data is shown in Fig. 13. CASSIOPE entered the conical wake region nearly 10 km behind the debris object (See Fig. 15). Broadband energy at frequencies less than 10 kHz did appear, but the proximity of the conjunction near the auroral oval (~ 70° latitude) where natural currents from Earth’s magnetosphere complicates the differentiation of this wave energy from the passing object. Channel B’s polarization also shows power below 6 kHz and is emphasized in Fig. 14 centered on the TCA of the conjunction. As this conjunction occurred within the auroral oval, the energy observed on Channel B may originate from the magnetosphere despite being correlated with the TCA. Some VLF energy below 6 kHz is visible but occurs ~ 3 s prior to the conjunction and approximately 16 s afterward (Fig. 15). The association is deemed to be medium-weak for this conjunction as it is unclear if it is associated with the object of the natural auroral background. As CASSIOPE passed through the far wake it is likely that any ion rarefactions would have merged with the nominal background ionosphere density.

### Iridium 911 (Medium-Weak)

**Fig. 16:**
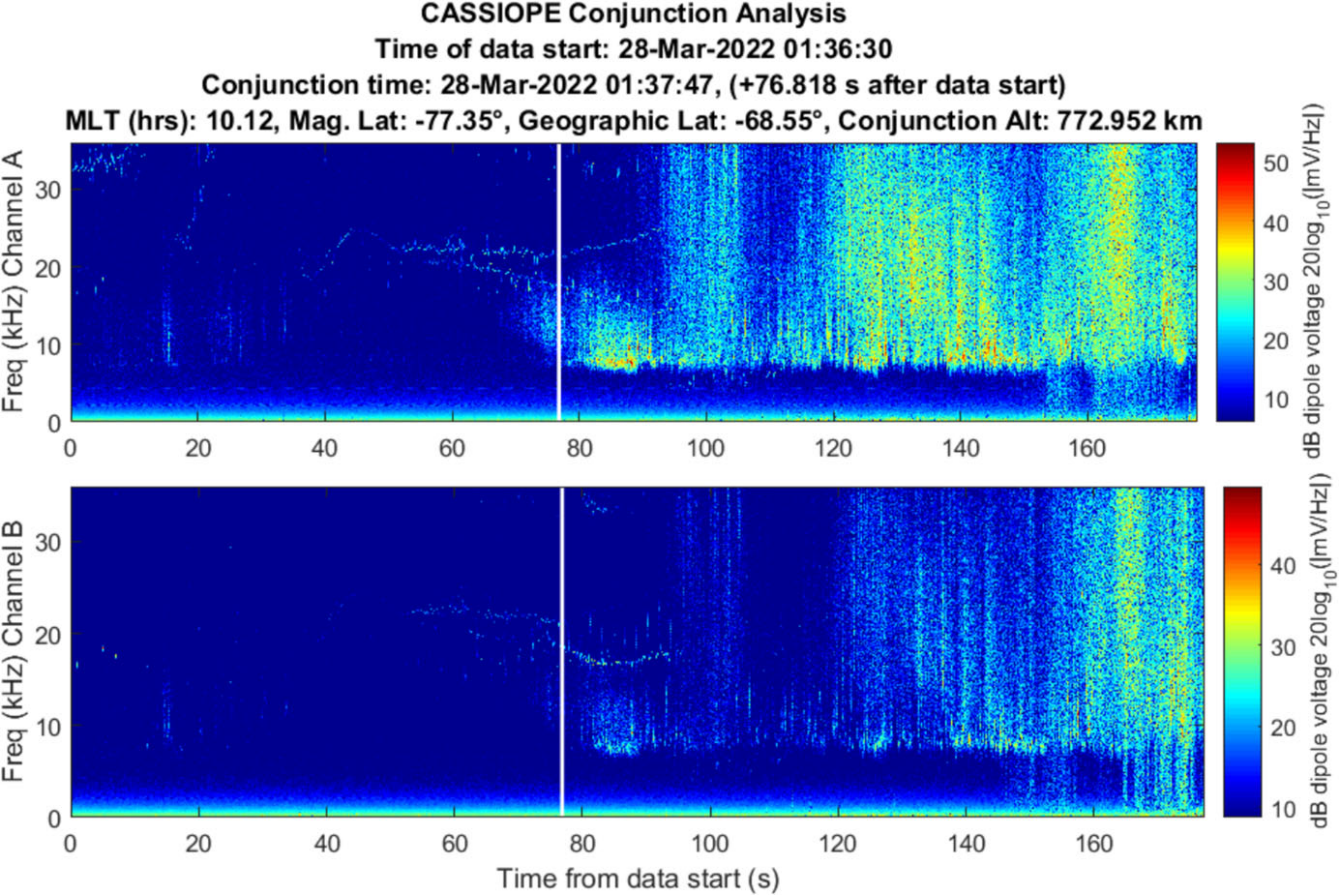
Spectrogram of detected VLF electric fields during CASSIOPE’s conjunction with Iridium 911

**Fig. 17:**
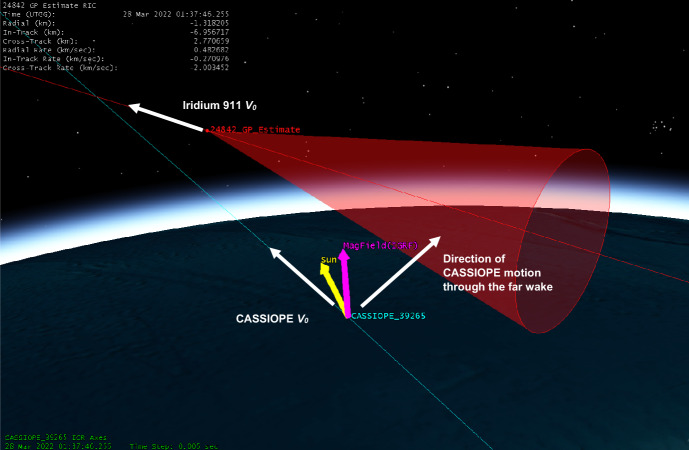
CASSIOPE’s motion relative to Iridium 911 showing its trajectory through the far wake of Iridium 911

CASSIOPE had a conjunction with Iridium 911 with a miss distance of 7.32 km with an in-track miss distance of 7.17 km. RRI data is shown in Fig. 16. Power above 6 kHz is visible but the location of the conjunction near the southern auroral region makes it difficult to attribute the detected wave power to the Iridium satellite. Significant energy is visible in Channel A which may be due to auroral particle precipitation. This conjunction was interesting in that CASSIOPE’s orbit was nearly parallel with the direction of motion of Iridium 911 and CASSIOPE took a full 2 s to traverse the 10° conical region behind the space object. CASSIOPE averaged ~ 7 km behind Iridium 911 during its 2 s passage though the extended wake region. While some wave power exists near the TCA it is assessed to be medium-weak due to the absence of broadband power at the time of conjunction and the relatively distant separation between CASSIOPE and Iridium 911 (Fig. 17).

### Other Conjunctions (Weak Association)

The other 31 conjunctions analyzed did not show power correlating in time with the conjunction TCA. This is largely attributed to these conjunctions’ larger stand-off distances or due to CASSIOPE passing far ahead of the wake region. The features observed in the first 2 Starlink encounters did not reappear during other Starlink passes observed in the dataset. Appendix 1 summarizes the findings for all collections and indicates if broadband power was observed near the TCA for the conjunction. Commentary on the presence of whistlers, audible voice recordings from VLF broadcasts, data interruptions and other phenomenology are noted.

## Interpretation

The conjunction geometry required for this experimentation was very challenging and very constraining for planning. Conjunctions where CASSIOPE passes through the ion acoustic wake of another space object are rare despite the generally high frequency of conjunctions in LEO. Given that most objects passed many kilometers from CASSIOPE, the data collected in this initial campaign makes it difficult to conclude if the sensing of a space object by detecting ion depletion (or enhancements) behind the moving space object is feasible. More data is currently being acquired, and this work represents a first initial attempt at sensing known space objects by looking for electric field oscillations in the background plasma environment. Electric field strengths detected during the four most promising conjunctions did not show fields anywhere near the 1 V/m expectation for wake traversals. The detected field strengths were closer to ~ 1 mV/m which is more consistent with the behavior of electrostatic noise [16].

The data acquired on Starlink 2672 and Starlink 2521 showed some indication that broadband energy detection may be detectable, but the lack of repeatability of the conjunction geometry and the significant time offset of Starlink 2672 makes these results inconclusive. The results may reinforce the prediction that the size scale of the ion depletion region “merging” back into the ambient density environment should do so at a distance of $z \sim 2\pi {V}_{0}^{2}/\Omega {\left({k}_{B}T/{m}_{i}\right)}^{1/2}$ for both species of dominant ions (O + and H +). Above 300 km altitude, this would be in-track distance scales of the ion acoustic wake of ~ 1.7 km for O + and 0.03 km for H + . This suggests this experimentation should focus on the LEO environment below 800 km where O + is the dominant ion where wake traversals are more likely to occur. This experimentation should also focus on collections on the dayside of the Earth where the diurnal O + ionospheric density exceeds that of H + . The broadband power fall-off expected from Eq. 8 was not exhibited in the Starlink 2571 data and may be simply because the refined orbital position of Starlink 2571 showed that it did not enter the wake region but rather the geomagnetic field “threaded” the Starlink’s wake. This may be indicative of Lower Hybrid Resonance plasma wave noise which is known to follow geomagnetic field lines (see Sect. 8).

The VLF noise environment posed challenges to disentangle VLF signals from nearby space objects in space. Artificial noise sources from ground-based VLF transmitters tend to operate above 15 kHz and their steady predictable tones do not appear to offer a great challenge when inspecting spectrograms for broadband VLF noise. However, the natural VLF environment comprised of whistlers and other auroral noise does generate considerable power in the frequency ranges expected for this experimentation.

Geometric complexity of the wake plays a role as well. The wake of a space object tends to broaden in the *x*-direction when the space object’s motion is perpendicular to the geomagnetic field. Therefore, an object is more likely to cross the wake when conjuncting with space objects at higher magnetic latitude. This is tempered by the fact that the auroral regions near ± 70° latitude strongly precipitate particles which could overwhelm subtle electric field measurements. The auroral regions, which tend to have a promising velocity-magnetic field geometry, pose a challenge to observe subtle ion density depletions behind a space object due to high natural auroral electron currents.

### Recommendations

Upon analysis of the RRI data, and assessment of the geometric and other space physics constraints to attempt detection of ion depletion regions in the wake of a space object, the following recommendations are made if follow-on attempts to detect ion density depletions are performed:

1. **Altitude:** The conjunction should be planned for altitudes less than 800 km which help ensure that O + is the dominant ion species. The heavier ion mass of O + increases the effective length of the wake before the density depletions merge back into the ambient ion density environment due to natural thermal motion of the ions.
2. **Dayside conjunctions:** Dayside ionospheric plasma is predominantly O + . Conjunctions where VLF data are collected should occur when the spacecraft and the ambient ionospheric plasma are dayside illuminated is recommended. This helps ensure the wake region is extended to help increase the likelihood of traversal.
3. **Conjunction planning:** While TLEs were used as the primary method to compute the TCA for the conjunction and the motion relative to the wake, the use of CDMs from the 19th Space Control Squadron is recommended for increased orbital planning precision. CDMs also contain useful ancillary data regarding the orbital uncertainties of the objects and the estimated radar cross section (size) of the secondary object. Alternatively, TLEs based on the operator ephemeris, such as the supplemental TLE available from Celestrak [12] can offer a better degree of precision for some constellations which publish operator ephemeris.
4. **Mach cone constraint:** The original planning approach for this experimentation used “any” conjunction to plan RRI data acquisition regardless is the object passed in front or behind of CASSIOPE. A further constraint where a ~ 7–10° half cone angle representing the Mach cone should be enforced to validate if the sensor passes through the Mach cone. The extent of the Mach cone should not exceed ~ 2 km in the wake direction of the space object. This will have the effect of significantly limiting the number of conjunctions but will produce encounter geometry which may be more likely to support the observation of ion density rarefactions.
5. **Latitude:** The auroral regions near ± 70° north and south latitude which have an extent of ~ 6–10° across should be avoided due to the likelihood of high background noise from magnetospheric currents in this region. Geomagnetic storms may further degrade acquisition effectiveness thus geomagnetically quiet conditions may be preferable for this research.
6. **Magnetic field measurements:** Electric field measurements were collected during this initial experimentation with CASSIOPE. It is recommended to collect magnetic field measurements to search for the presence of plasma waves where magnetic field oscillation modes may be observed. While it is not expected that the induced magnetic field due to a charged space object motion would likely be detectable in LEO (as indicated in Sect. 2), other field-aligned phenomena may be possible avenues to explore.
7. **Orbital analysis:** CASSIOPE’s GPS ephemerides should be relied upon more heavily for orbital analysis as they provide more accurate positioning of the RRI relative to the wake of another space object. Secondary objects in a conjunction are likely to be either debris (having no operator ephemerides) or Starlink satellites (where operator ephemeris is sometimes available). CDM messages are a better product to use in the relative orbit analysis of debris objects as operator ephemerides are unavailable. CDMs also have improved position accuracy in comparison to two-line orbital elements and quantify the position uncertainty at TCA. Given the narrow geometry of the wake from 0.25 – 1.5 km the combination of observer GPS ephemeris and CDM data provide better orbit information to assist relative motion analysis.
8. **Co-orbital relative motion**: Only one conjunction in this study had relative motion geometry where CASSIOPE spent ~ 2 s traversing the Mach cone wake of the primary satellite. This near co-orbital motion did not show the hoped-for signature of broadband noise and was likely due to the ~ 7.3 km gap from Iridium 911 and CASSIOPE. As many satellites are now launched simultaneously as upper stage “ride-shares” a satellite equipped with a Langmuir probe or a VLF receiver and with a modest degree of maneuvering capability could attempt measuring the wake region of another satellite co-orbitally. This would provide an additional control for the conjunction geometry and possible entry into the near wake of another satellite. This is an avenue which could be explored if a suitable space-physics package is launched with other satellites to orbit.

## Future Direction of Research

It has been long known that plasma wave noise can be self-generated by LEO spacecraft [16] and interplanetary spacecraft during encounters with the Earth [17]. Recent experimentation has found that thruster bursts from space objects in LEO where a considerable amount of propellant is used can be detected several hundred kilometres from the maneuvering satellite as electric field noise [18]. The sensing of propulsion events would be useful to the SSA community as it would provide direct insight as to whether a satellite was maneuvering at the time of observation. This circumvents the necessity of measuring large orbital arcs using classical space surveillance approaches and determining if orbit determination residuals show evidence of a maneuver. Direct measurements indicating if a satellite thruster was in use can provide immediate insight into the operational state of a satellite.

Formation flight measurements from a small subsatellite performing elliptical relative motion about space shuttle Atlantis in 1988 [16] detected broadband electrostatic noise a) within the wake of the shuttle, and b) during intervals where geomagnetic field lines “threaded” the shuttle’s wake. This noise was detected at ranges up to several hundred meters from the shuttle. The noise was attributed to currents stimulated by a cloud of water vapor and other particles emitted by the shuttle either venting or maneuvering in space. The noise was also uniquely detected at times when the local magnetic field line “threaded” the wake of the space shuttle even though the subsatellite was clearly outside of the wake region. This suggests that field-aligned waves may be detectable if the noise generation source originated in the wake of the space shuttle.

During the Jupiter-bound Galileo mission’s gravity assisted pass by the Earth, its electric field sensor noted periodic pulses in the Lower Hybrid Resonance (~ 6 kHz) [17] which were found to be attributed to the spin rate of Galileo, and the times that its boom-mounted electric field antenna threaded a magnetic field line with Galileo’s own wake. The LHR noise was attributed to be the origin of those waves, possibly due to the Lower Hybrid Mode drift instability [17].

Both the findings from the shuttle, and that of Galileo suggest that electrostatic noise propagating along geomagnetic field lines may be another avenue to explore to sense the presence of space objects. Both allude to noise which propagates along the geomagnetic field lines and is suggestive of the lower hybrid mode – a mix of ion and electron gyrofrequencies and the plasma frequency when wave propagation is nearly perpendicular to the magnetic field. While the Starlink 2521 findings were not repeated during the other measurements in CASSIOPE’s experimentation, the geometric condition of its observation was consistent with these two historical LHR findings. Alternatively, propulsion-related activity cannot be ruled out as propulsion events are known to generate significant electric field perturbations at long distances from spacecraft [18]. Starlinks regularly operate electric propulsion systems, therefore an examination of the emitted signatures of these systems should be considered.

Current work is now focusing on plasma wave generation mechanisms from charged space objects in LEO. Space objects are predicted to charge slightly negative (~ -0.5 V) and may generate plasma waves. This may offer another approach to sense space objects aside from charge density variations attempted in this campaign. This is now being explored as an alternative means to detect space objects at potentially greater distances, and much more enabling geometries for a space-based observer [19].

## Conclusion

CASSIOPE collected space-based observations of the VLF environment during conjunctions between itself and other space objects. The aim was to detect broadband wave energy provoked by CASSIOPE’s rapid traversal of ion density rarefactions in, or near, the ion-acoustic wake of another space object. The rigid geometric constraints required for this type of observation, and the generally large separations between CASSIOPE and other space objects yielded very limited data suggesting that density rarefactions is a possible approach to observe the presence of a space object. It does not appear that traversal of satellite wakes and measuring ion density rarefactions is a viable means to sense the presence of another space object.

The strongest evidence of detection was demonstrated in one conjunction between Starlink 2571 where CASSIOPE passed below the Starlink, but its wake region connected to CASSIOPE via geomagnetic field lines. Broadband energy spanning the ion gyrofrequency to the Lower Hybrid Resonance (~ 6 kHz) was observed during this encounter. Another conjunction between Starlink 2672 similarly showed broadband energy near the Lower Hybrid Resonance but was observed ~ 63 s prior to TCA. This suggests the detected wave power may not have been associated with Starlink 2672 but perhaps another object or electric thrust transients from the Starlink constellation. A conjunction between COSMOS 2251 debris and Iridium 911 showed VLF power enhancement at TCA below 6 kHz but were difficult to differentiate from natural ionospheric and auroral environment effects. All other 31 conjunction observations did not yield low frequency VLF power associated with their TCA or were instances where CASSIOPE traversed the wake of the secondary object. Repeatable, clear signatures of space object passage were not observed in the VLF data collected during this campaign.

Detected electric field strengths were in the ~ 1 mV/m range or less and did not reach anywhere near the ~ 1 V/m range predicted for a wake traversal. CASSIOPE passages through the far wake (> 6 km) of some space objects (COSMOS 2251 debris, and Iridium 911) showed VLF electric field strengths less than ~ 1 mV/m.

This work did highlight several geophysical and orbital constraints which could help refine time and geometry to observe conjunctions. Future work will use more rigorous constraints when planning RRI data acquisition respecting the quasi-conical geometry of the wake, space object altitude and the auroral regions. Plasma wave approaches will be examined in the future as an alternative means to detect space objects by searching for charged particle current, magnetic or electric field oscillations. It is now believed that VLF waves spanning the ion cyclotron frequencies (~ 0.036—2 kHz) to the Lower Hybrid Resonance (~ 6 kHz) appears to be a key phenomenology of space plasma physics for objects moving at high relative velocities in Earth’s ionosphere.

## Data Availability

All RRI data collected from this campaign can be openly downloaded from the University of Calgary’s e-POP data server https://epop.phys.ucalgary.ca/ [Also see reference 11].

## Appendix 1: Secondary objects conjuncting with CASSIOPE in 2022 where RRI data was collected

**Table:**

SSN ID	Object Name	Time of Closest Approach (UTC)	Miss distance (km)	Secondary object altitude (km)	Radial miss (km)	In-track miss distance (km)	Cross-track miss distance (km)	Magnetic latitude (deg)	MLT (hrs)	Sun Cond	VLF power observed at TCA? / Notes
48,650	STARLINK-2672	04 Mar 2022 00:28:12.162	17.73	554.50	1.30	− 7.71	− 15.91	− 29.28,	2.18	Eclipse	TCA—63 s
48,648	STARLINK-2521	04 Mar 2022 03:48:43.179	0.24	555.28	0.09	− 0.09	− 0.20	− 21.00,	2.30	Eclipse	At TCA
35,671	COSMOS 2251 DEB	27 Mar 2022 08:08:00.326	7.92	782.44	0.16	5.22	5.95	68.87	19.79	Sun	At TCA, auroral noise
24,842	IRIDIUM 911	28 Mar 2022 01:37:47.249	7.32	773.07	0.83	7.17	1.19	77.35	10.12	Sun	Auroral noise after TCA
41,416	NOAA 16 DEB	06 Apr 2022 04:31:24.390	4.78	839.15	− 3.04	− 0.54	− 3.65	− 74.97	1.97	Sun	–
30,680	FENGYUN 1C DEB	06 Apr 2022 11:13:12.202	4.01	863.56	− 1.23	0.44	3.79	− 71.33	5.67	Sun	–
41,950	FLOCK 3P 20	06 Apr 2022 21:00:44.617	4.82	482.06	2.82	− 0.15	− 3.91	− 39.97	21.91	Eclipse	–
41,064	NOAA 16 DEB	06 Apr 2022 21:14:29.954	2.69	861.43	1.48	0.48	2.19	− 89.55	11.42	Sun	–
30,237	FENGYUN 1C DEB	07 Apr 2022 08:55:15.654	1.85	838.14	0.17	0.05	1.84	− 71.59	3.29	Sun	Data gaps during recording
47,520	FLOCK 4S 29	07 Apr 2022 11:35:03.538	4.78	539.13	− 4.68	− 0.17	− 0.93	61.45	20.56	Sun	–
47,442	SPACEBEE-63	07 Apr 2022 14:55:43.828	3.81	533.71	3.73	0.14	0.75	− 43.06	21.80	Sun	Strong whistlers at TCA
30,054	FENGYUN 1C DEB	07 Apr 2022 18:56:50.673	2.53	844.28	1.48	− 0.44	2.00	− 89.35	5.67	Sun	Auroral noise
48,372	STARLINK-2572	08 Apr 2022 23:11:20.270	95.30	576.63	− 12.23	− 84.71	− 41.93	− 43.06	21.80	Eclipse	Strong natural whistlers
44,084	LEMUR 2	09 Apr 2022 07:28:40.736	7.05	479.87	− 2.13	− 0.06	− 6.73	− 29.67	22.23	Eclipse	–
47,426	SPACEBEE-46	09 Apr 2022 12:31:41.079	6.15	537.58	− 6.09	0.14	− 0.85	− 48.77	22.31	Eclipse	B–field threads wake, whistlers
29,956	FENGYUN 1C DEB	09 Apr 2022 12:44:26.985	8.94	899.11	3.10	− 1.58	8.23	− 75.0	5.32	Sun	–
25,262	IRIDIUM 51	12 Apr 2022 01:47:28.806	11.81	596.97	3.96	− 7.38	8.33	73.89	11.33	Sun	–
20,510	OKEAN 2	12 Apr 2022 08:28:54.956	8.38	582.04	− 7.92	2.14	− 1.72	71.67	14.66	Sun	Data gaps in h5 file
48,021	STARLINK-2301	12 Apr 2022 09:01:46.299	16.28	563.02	− 3.50	− 7.84	− 13.83	− 38.20	21.99	Eclipse	Whistlers near TCA
22,591	SL-8 R/B	12 Apr 2022 09:54:34.915	12.28	970.20	− 11.00	− 1.26	5.31	37.17	9.85	Sun	Audible voice broadcast?
52,111	STARLINK-3708	12 Apr 2022 10:20:35.789	236.10	354.01	− 3.84	− 210.25	− 107.36	40.30	19.96	Sunset	High noise environment
47,979	STARLINK-2325	12 Apr 2022 12:22:14.031	16.78	563.64	− 4.07	− 7.98	− 14.19	− 44.87	21.73	Eclipse	Heavy whistlers, 20 kHz noise
44,820	CZ-4C R/B	12 Apr 2022 15:09:58.807	15.44	576.79	14.90	− 0.12	4.03	81.73	17.86	Sun	–
47,765	STARLINK-2183	12 Apr 2022 20:24:02.644	189.37	334.10	4.69	95.52	163.45	41.78	21.16	Sunset	Heavy noise at TCA
11,060	TIROS N	12 Apr 2022 22:33:16.183	19.02	844.50	7.81	0.00	17.34	− 66.03	21.45	Sunrise	Heavy auroral noise
52,103	STARLINK-3705	13 Apr 2022 21:26:52.446	350.96	337.65	5.67	− 316.93	− 150.66	49.18	20.99	Sunset	Audible voice broadcast?
45,076	STARLINK-1180	13 Apr 2022 23:26:03.625	15.91	543.80	12.82	− 4.62	− 8.21	− 22.98	21.15	Eclipse	Whistlers at TCA and about
45,139	ONEWEB-0026	14 Apr 2022 04:52:03.893	485.27	1175.24	52.37	481.37	31.83	−	−	–	Recording begins 8.5 s after TCA
49,215	ONEWEB-0351	14 Apr 2022 07:16:51.903	87.17	532.69	77.54	− 27.38	28.92	71.49	14.2	Sun	–
51,625	ONEWEB-0416	14 Apr 2022 10:25:31.506	89.23	847.38	− 60.61	− 59.65	27.03	48.58	9.91	Sun	–
49,298	ONEWEB-0372	14 Apr 2022 10:36:23.435	60.83	554.85	49.21	− 19.88	29.73	74.27	14.99	Sun	–
45,348	SL-14 DEB	25 Apr 2022 16:40:59.056	5.44	1198.32	0.36	− 1.09	5.32	− 40.70	7.29	Sun	No recording at TCA
48,055	ONEWEB-0157	30 Apr 2022 05:03:18.579	6.77	1238.80	0.35	− 5.99	− 3.14	− 71.64	1.14	Sun	–
46,027	STARLINK-1522	09 Jun 2022 10:47:58.872	3.07	550.59	0.54	1.72	− 2.48	48.49	0.96	Sun	–
31,126	MAST	10 Jun 2022 04:33:30.934	0.21	668.50	− 0.03	− 0.02	− 0.20	− 77.71	19.41	Sun	Electronic noise at TCA

Shaded rows indicate conjunctions where some broadband power was observed in the VLF and associated with the time of conjunction

## Footnotes

1 All RRI data collected from this campaign can be openly downloaded from the University of Calgary’s e-POP data server https://epop.phys.ucalgary.ca/ [11]
